# Enhancing the Thermal Performance of Shape Memory Polymers: Designing a Minichannel Structure

**DOI:** 10.3390/polym16040500

**Published:** 2024-02-11

**Authors:** Saed Beshkoofe, Majid Baniassadi, Alireza Mahdavi Nejad, Azadeh Sheidaei, Mostafa Baghani

**Affiliations:** 1 School of Mechanical Engineering, College of Engineering, University of Tehran, Tehran 1439814151, Iran; 2 School of Engineering, Wentworth Institute of Technology, Boston, MA 02115, USA; mahdavinejada@wit.edu; 3 Aerospace Engineering Department, Iowa State University, Ames, IA 50011, USA

**Keywords:** thermal performance enhancement, shape memory polymer, minichannel networks, fluid–structure interaction, thermo-mechanical properties

## Abstract

This research proposes a numerical approach to improve the thermal performance of shape memory polymers (SMPs) while their mechanical properties remain intact. Sixteen different 3D minichannel structures were numerically designed to investigate the impact of embedded water flow in microchannel networks on the thermal response and shape recovery of SMPs. This work employs two approaches, each with different physics: approach A focuses on solid mechanics analysis and, accordingly, thermal analysis in solids without considering the fluid. approach B tackles solid and fluid mechanics analysis and thermal analysis in both solid and fluid subdomains, which inherently calls for fluid–structure coupling in a uniform procedure. Finally, the results of these two approaches are compared to predict the SMP’s thermal and mechanical behavior. The structural designs are then analyzed in terms of their shape recovery speed, recovery ratio, and recovery parameters. The results indicate that isotropic structures thermally outperform their anisotropic counterparts, exhibiting improved thermal characteristics and faster shape recovery. Additionally, it was observed that polymeric structures with a low volume fraction of embedded branches thermally perform efficiently. The findings of this study predict that the geometrical angle between the main branch and sub-branches of SMP favorably impacts the enhancement of thermal characteristics of the structure, accelerating its shape recovery. Approach B accelerates the shape recovery rate in SMPs due to fluid flow and uniform heat transfer within the structures.

## 1. Introduction

Smart material comprises a class of materials capable of responding to exterior stimuli like temperature, pH, chemical compounds, or electric fields in a controlled and irreversible way [1]. Several studies have been conducted concerning shape memory polymers (SMPs) in the area of smart materials. SMPs maintain the advantages of low density, low material/fabrication price, considerable shape recovery, and easy manufacture and programming [2]. They can return from a temporarily deformed state and recover their original shape when induced by outer stimuli, such as heat, pH, microwaves, light, electric field, or focused ultrasound [3]. However, the most common stimulus is temperature variation since it can be applied easily compared to other stimuli. Also, SMPs based on thermal response, due to their excellent shape recovery performance, are well-suited for biodegradable fields, including drug delivery, airway and artery stents [4], and tissue engineering [5]. As an example, with practical interest, SMPs offer minimally invasive surgeries and exhibit an enormous potential in vascular and tracheal stents [6]. Furthermore, if the recoverable ability of SMPs is incorporated into a microfluidic system, the flow in the channel can be programmed [7]. Moreover, they have multiple potential applications in sensors, valves [8], and intelligent devices [9,10,11]. The intelligent control of the fluid flow in a multi-channel is one of the main reasons for using SMPs in heat sink devices. Microchannel heat sinks are space-constrained electronic devices and are considered the most effective heat removal devices [12]. The SMP can drive fluid reversibly through a microfluidic channel and then pump it in response to environmental temperature changes [13]. Similarly, developing an active microfluidic reservoir by employing SMPs was accomplished in [7,14].

Different parameters affect the behavior of SMPs, such as geometrical dimensions [15], the loading type, stimuli type, material distribution [16], and mechanical loading rate [17]. Thus, employing an appropriate 3D constitutive model is important to simulate different SMP structures [18]. According to the models available in the literature, two approaches have been primarily embraced: the phase transition and thermo-viscoelastic approaches. The phase transition approach represents shape memory as a transformable phase that can transform rubbery and glassy phases into another [19]. Compared with models based on phase transition models, the viscoelastic model does not physically describe the SME of the glass transition. Tobushi et al. [20] pioneered the SMP constitutive model, adjusting a four-element linear viscoelastic model, which describes the deformation properties of SMPs. Diani et al. [21] derived a 3D thermo-viscoelastic constitutive model based on the viscoelastic properties of cross-linked SMP networks by considering the large strain deformation. A multi-branch model that utilizes two sets of non-equilibrium branches for SMP’s phases was presented to arrest the SME by evaluating the properties of amorphous SMPs [22]. Based on this idea, Diani et al. indicated the amorphous polymer network’s shape recovery response by employing dynamic mechanical analysis (DMA) to measure the viscoelastic behavior of an amorphous SMP and its time and temperature reliance [21,23].

In order to modify the SME of smart polymers, altering the geometry of the material’s structure and adapting rational design can be used [24]. Porous SMP, which indicates geometric and volumetric change by outer stimuli such as heat transfer, can be made into foams, meshes, biomechanical devices, and heat sinks [25]. The geometrical design of SMPs allows comparable or better properties to be achieved for required applications [16]. Previous studies found that the dimensional and geometrical design characteristics of SMPs significantly impact their ability to control their shape recovery because geometrical designs and material responsiveness influence the transition rate. Recently, the power density of electronic chips has been developing remarkably due to their shrinking dimensions. Enormous efforts have been made to optimize heat sinks’ geometric design and improve the thermal performance of microchannel heat sinks (MCHSs) by a specific volume [26]. Bejan et al. [27] originally represented heat sinks by inspiring nature’s transport systems, e.g., plant roots and animal respiratory systems. They suggested that the heat sinks be made of tree-shaped microchannels with fractal networks that allow for higher transport efficiency, lower flow resistance, and an improved uniform temperature.

In this research, various 3D solid–fluid-conjugate heat sinks made of SMPs are innovatively proposed. A numerical study was performed by employing the microchannel models reported by Wang et al. [28] to examine the effect of heat transfer in SMP microchannel networks and its impact on response time and shape recovery for polymeric structures. This study employs two distinct approaches. Approach A centers on the investigation of solid mechanics and heat transfer in solid materials without the involvement of fluid dynamics. Meanwhile, approach B incorporates solid mechanics, laminar flow, and heat transfer into solid and fluid physics, necessitating solid and fluid dynamics coupling in a unified process. As such, each approach utilizes disparate physical principles, and the results obtained from both approaches are compared to predict the thermal and mechanical properties of SMPs.

Furthermore, an appropriate nonlinear constitutive model predicts SMP behavior in a multi-physics framework. This model is applied in COMSOL multi-physics software, enabling the multi-physics analysis of systems, including SMP components. The following two classes of geometry designs are studied: anisotropic and isotropic models. This analysis eventually aims to achieve the most efficient designed model with optimized SMP properties and the quickest response time and shape recovery. In fact, by inspiring the heat sink topology, SMP structures were designed to enhance the thermal properties and shape memory effect in the shape recovery and response time of SMPs.

## 2. Material and Model

In this research, the SMP structures were designed to minimize the response time to thermal stimuli. It is crucial to note that heat convection significantly influences SMP behavior within a multi-physics framework. This study explores the different design types of SMP structures and discusses the modeling of their behavior. It also covers two approaches used to predict SMP behavior in a fluid and solid-coupled system and the numerical method employed to investigate their thermal and mechanical behavior.

### 2.1. Designing the Structure

**Figure 1:**
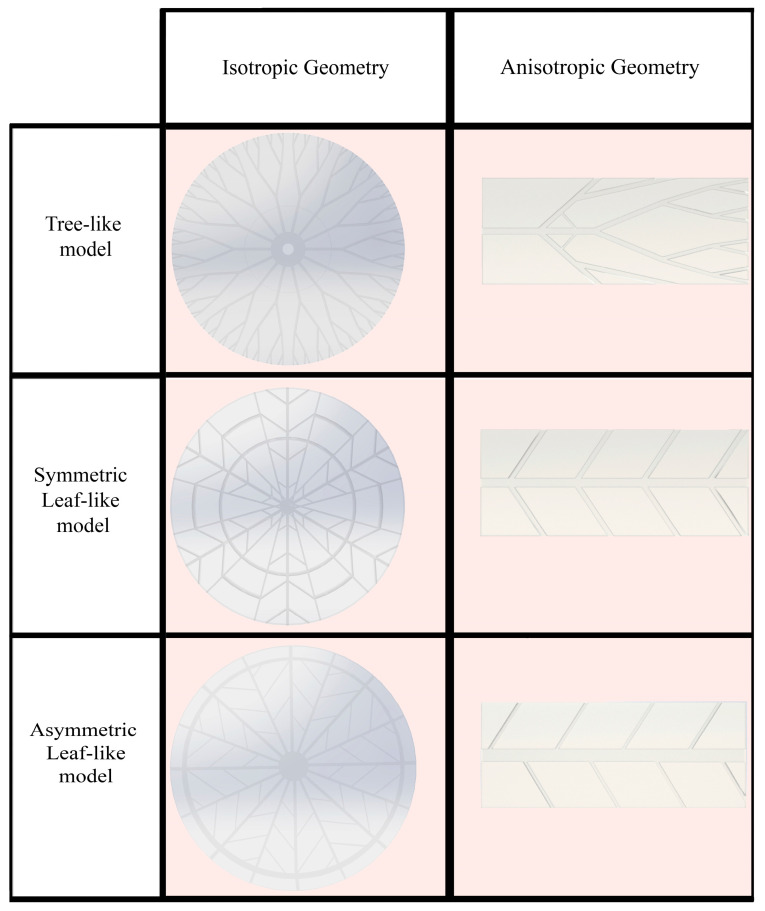
The two main 3D-designed structures: isotropic and anisotropic geometry.

**Figure 2:**
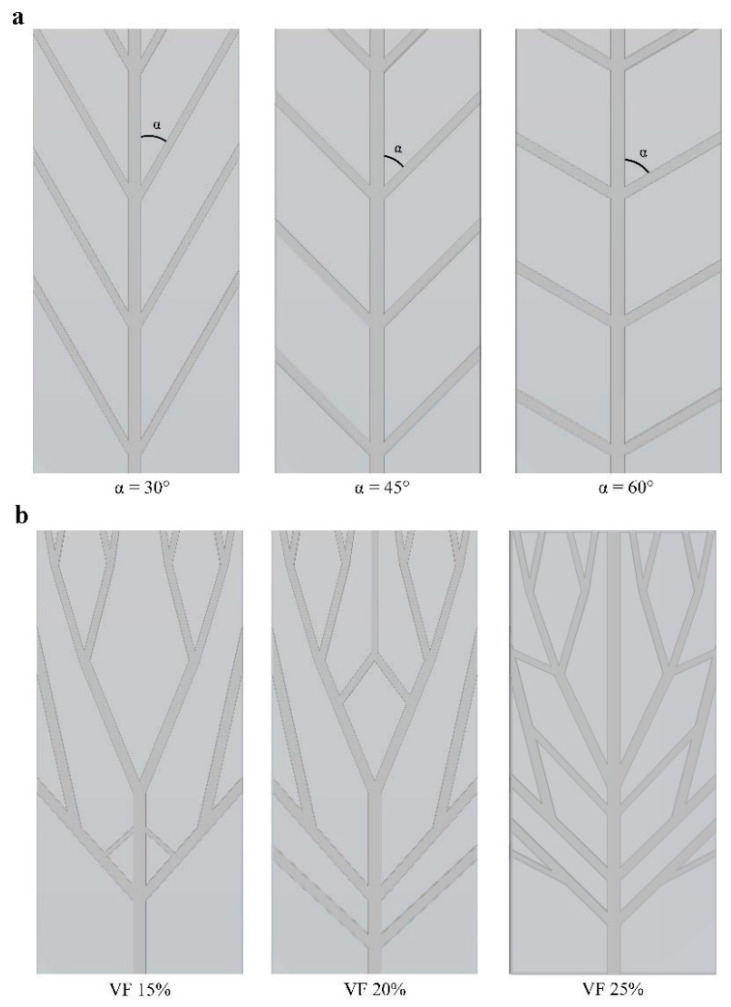
(**a**) Three different angles in designed structures (for example, the symmetric leaf-like structure). Three angles are designed in symmetric and asymmetric types of structures. Also, each structure with 45 angles has three different VFs. (**b**) Three VFs of branches in a tree-like anisotropic structure.

The escalating need for faster performance in electronic devices has recently posed a considerable challenge [29]. High performance generates excess heat, causing devices to overheat and malfunction. MCHSs, which operate relatively straightforwardly, were introduced to enhance heat removal from integrated circuits [30]. The generated heat of electronic equipment is transferred to the coolant fluid using the convection mode of heat transfer. In this case, by designing heat sinks made of SMP, an attempt is made to pronounce the cooling process. Also, these structures can improve the thermal properties of SMPs. This research aims to reduce the shape recovery time and, thus, improve the response of the SMP structure to heat. Two types of structures were designed: isotropic and anisotropic structures. As shown in Figure 1, anisotropic structures have a rectangular shape as well as unevenly distributed heat and fluid flow, and isotropic structures have a circular shape in which fluid flow and heat are uniformly distributed. Depending on the preferred cooling application, any designed structures can be used. As mentioned, the initial design idea was based on the plant’s water supply system. The models’ representative volume element (RVE) was based on the structure dimensions represented by Wang et al. [28]. As shown in Figure 2, the geometry of the models is divided into three general types: tree-like, symmetric leaf-like, and asymmetric leaf-like, and each model is designed by three volume fractions (VFs) of embedded branches: 15%, 20%, and 25%. The asymmetric and symmetric models were designed with three angles: 30°, 45°, and 60°. As Wang et al. [28] suggested, some geometrical parameters need to be defined for the design of networks to obtain an appropriate comparison. The two following conditions were utilized:

- The total surface area exposed to convective heat transfer fluid is fixed for each flow network.
- The inlet cross-section area at the loading step is the same for various structures.

All anisotropic structures are designed as 20 × 8 × 2 mm cubes.

This research uses the following two approaches: approach A focuses on solid mechanics analysis and heat conduction in solids without fluid. Approach B still employs solid mechanics analysis and heat transfer in solids but in the presence of a coolant fluid, which requires structure–fluid coupling in a uniform procedure. It implies that the coupling of solid and fluid mechanics is consistent and systematic throughout the analysis. Therefore, each approach deals with different physics, which are compared to evaluate how accurately they predict SMP behavior. The following subsections explain the SMP constitutive equation and its relevant boundary conditions for each approach individually.

### 2.2. Structural Model of the SMPs

As Bakhtiyari et al. [31] suggested, all SMP-designed MCHSs are incompressible and isotropic hyperelastic models. The neo-Hookean hyperelastic model was utilized to fit the average stress–strain curve and determine the coefficients applicable to SMP-designed models. The strain energy density function (SEDF) for this model is written as follows:

(1) $$\Psi =C_{1}(I_{1}-3)$$

where $C_{1}$ represent material constant coefficients, and *I*_1_ is the first invariant. Furthermore, the large strain viscoelasticity theory proposed by Holzapfel [32,33] is implemented. The strain energy in the generalized Maxwell equation is decomposed into two parts: volumetric and isochoric parts. The branches of viscoelasticity contribute to the following equation:

(2) $$W=W_{vol}^{\infty }+W_{iso}^{\infty }+\sum_{\varsigma =1}^{m}\Upsilon_{\varsigma },$$

where in the main hyperelastic branch $W_{vol}^{\infty }$ and $W_{iso}^{\infty }$ are the strain–energy functions (the superscript ∞ denotes the long-term equilibrium). The viscoelastic contribution of the ς th branch represents the third term $\sum_{\varsigma =1}^{m}\Upsilon_{\varsigma }$. The second Piola–Kirchhoff stress is evaluated as follows:

(3) $$S=2\frac{\partial W}{\partial C_{M}}=S_{vol}^{\infty }+S_{iso}^{\infty }+\sum_{\;\varsigma =1}^{m}Q_{\;\varsigma }\;,$$

where $Q_{\varsigma }$ denote to the auxiliary second Piola–Kirchhoff stress tensor expressed as $Q_{\varsigma }=2\frac{\partial \Upsilon_{\;\varsigma }}{\partial C_{M}}$. The mechanical right Cauchy–Green tensor is $C_{M}=F_{M}^{T}F_{M}$. The auxiliary stress tensor’s time evolution is computed in every viscoelastic branch by the following:

(4) $$\frac{dQ_{\varsigma }}{dt}+\frac{Q_{\varsigma }}{\tau_{\varsigma }}=\frac{dS_{iso,\varsigma \;}}{dt},\;\varsigma =1,\;2,\;\ldots ,\;m,\;$$

where $\tau_{\varsigma }\in (0,\infty )$ indicates the relaxation time interrelated to the viscoelastic branch ς. $S_{iso,\varsigma }$ represents the isochoric second Piola–Kirchhoff stress tensor in the branch derived from the energy factor χ∈(0,∞). In the main hyperelastic branch, the strain energy density is as follows:

(5) $$W_{iso,}\varsigma =\chi_{\varsigma }W_{iso}^{\infty }\Rightarrow S_{iso,\varsigma }=2\frac{\partial W_{iso,\varsigma }}{\partial C_{M}}=2\chi_{\varsigma }\frac{\partial W_{iso}^{\infty }}{\partial C_{M}}=\chi_{\varsigma }S_{iso}^{\infty }.$$

Involving the convolution integrals, a closed-form solution for the linear evolution Equation (4) in the time interval t ∈ (0, T] can be calculated as follows:

(6) $$Q_{\varsigma }=\int_{0}^{t=T}exp\left(-\frac{T-t}{\tau_{\varsigma }}\right)\frac{d}{dt}\left(S_{iso,\varsigma }\right)dt.$$

Employing an update algorithm, the total stress in an FE framework can be expressed as follows:

(7) Sn+1=Siso∞+Svol∞+∑ς=1mQς n+1  

where

(8) $$\left\{\begin{array}{c} S_{vol,n+1}^{\infty }=J_{n+1}\frac{\partial {\psi \;}_{vol}^{\infty }}{\partial J_{n+1}}C_{Mn+1}^{-1}\;S_{iso,n+1}^{\infty }=2\frac{\partial {\psi \;}_{iso}^{\infty }}{\partial C_{Mn+1}},\\ Q_{\varsigma ,n+1}=exp\left(-\frac{\Delta t}{\tau_{\varsigma }}\right)Q_{\varsigma ,n}+exp\left(-\frac{\Delta t}{{2\tau }_{\varsigma }}\right)\chi_{\varsigma }\left(S_{iso,n+1}^{\infty }-S_{iso,n}^{\infty }\right). \end{array}\right.$$

The time–temperature superposition principle (TTSP) should apply when considering temperature changes in a shape memory effect. For this aim, William–Landel–Ferry (WLF) relations were used, which modify the time scale as follows [34]:

(9) $$\log_{10}A_{T}=-\frac{c_{1}(T-T_{ref})}{c_{2}+(T+T_{ref})}$$

where $c_{1}$, $c_{2}$, and $T_{ref}$ are material parameters, and $t_{r}$ is the reduced time defined as follows:

(10) $$t_{r}\left(t\right)=\int_{0}^{t}\frac{d\eta }{A_{T(\eta )}}$$

Thus, it is necessary to consider the TTSP in the cooling or heating procedure and apply the effect of temperature change to the problem by defining the reduced time. The material parameters of SMP are based on Arrieta’s work [35] which comprises viscoelastic, hyperelastic, TTSP, and thermal expansion properties. This material is an epoxy SMP, whose material parameters are detailed in Table 1. COMSOL multi-physics software is employed to numerically solve the strength and precision of SMPs.

**Figure 3:**
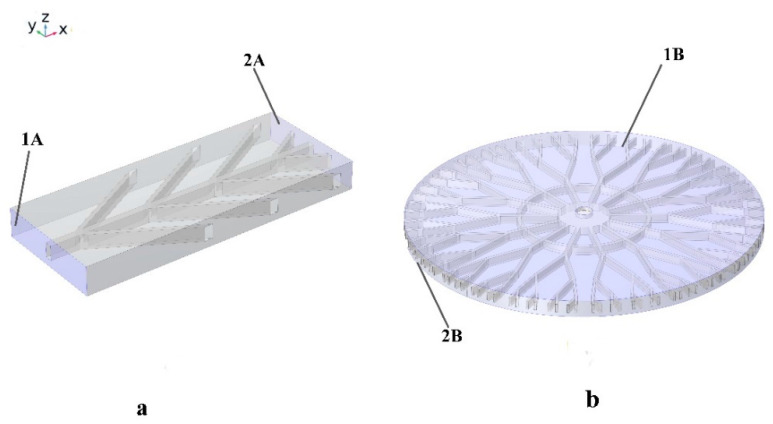
Mechanical boundary conditions for (**a**) anisotropic structures and (**b**) isotropic structures. Both 1A and 1B surfaces are considered fixed, and 2A and 2B are subjected to 3% tensile pre-strain.

Figure 3 exhibits how surfaces 1A and 1B are fixed, while 2A and 2B undergo axial loading to reach the 3% strain. For the meshing of SMP structures, the free triangular is chosen. Fifteen thousand elements were used to solve the problem, while 30,000 mesh elements were used for isotropic geometry.

### 2.3. Fluid-Structure Interaction- SMP Model Coupling

**Figure 4:**
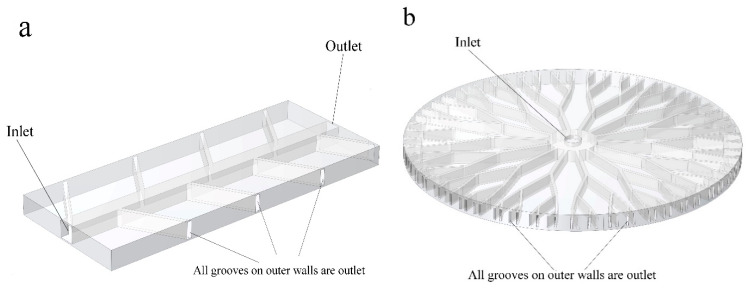
Fluid flow inlet and outlet; (**a**) Fluid path in anisotropic SMP structure and the (**b**) fluid path in isotropic SMP structure. All outer grooves are outlets.

**Figure 5:**
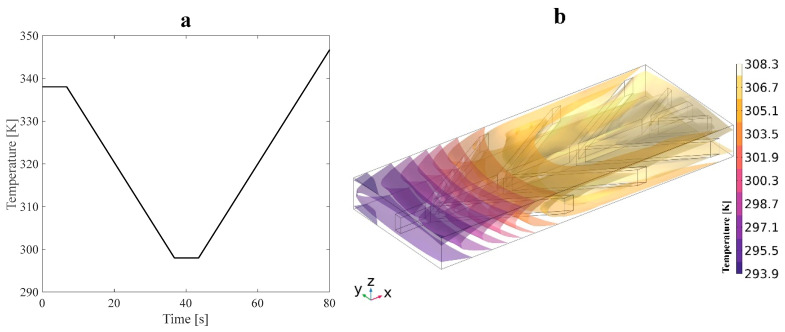
(**a**) Temperature profile and (**b**) isothermal contours induced by an applied heat flux during the cooling stage on the structure.

All 16 structures must be numerically studied to achieve the most accurate model based on mechanical properties and the shortest response time for SMEs. For an SMP structure introduced in Figure 4, heat transfer from the fluid flow to the structure and its effect on the SMP behavior is studied. Finite Element Method is employed to discretize equations governing both fluid flow and structural mechanics. The software used in this study is COMSOL Multi-physics 6. Additionally, the fluid flow in microchannel branches and its interaction with the solid matrix must be appropriately coupled to simulate the intelligent system. The fluid in the networks of the designed structures is assumed to be water and temperature-dependent thermos-physical properties, as follows:

The continuity, momentum, and energy equation of incompressible and laminar flow are as follows:

(11) $$\rho \nabla .u_{fluid}=0$$

(12) $$\rho \frac{\partial u_{fluid}}{\partial t}+\rho u_{fluid}.\nabla \left(u_{fluid}\right)=-\nabla p+\nabla .\left(\mu \left(\nabla u_{fluid}+\nabla {u^{T}}_{fluid}\right)\right)+F$$

(13) $$\rho C_{p}\frac{\partial T}{\partial t}+\rho C_{p}u_{fluid}.\nabla T+\nabla .\left(\lambda \nabla T\right)=Q$$

where ρ, λ, μ, and $C_{p}$ are the temperature-dependent fluid properties described in Table 2. Here, **F** and Q represent the external force and heat generation applied to the fluid, respectively. Equations (11) to (13) are solved for the fluid velocity field u, fluid pressure p, and fluid temperature, *T*. Approach B uses 40,000 and 150,000 elements for anisotropic and isotropic structures, respectively. The tetrahedral mesh type is used for the fluid subdomain. First, an external mechanical tensile load is applied to the structure. When the fluid temperature is raised, a temperature gradient in the SMP is induced, which causes the SMP to revert to its initial shape. Also, to simulate the shape recovery of the structure due to fluid temperature, the fluid–structure interaction (FSI) module is employed. A pressure drop was created by setting the inlet boundary condition to 5 Pa and the outlet boundary condition to 0 Pa. The temperature profile applied to the water was modeled as a linear function, with a constant temperature gradient along the length of the microchannel networks. The temperature profile is shown in Figure 5a. The heat transfer across the interface between the solid and fluid domains was modeled using a convective boundary condition. The convective boundary condition considers the heat transfer due to the movement of the fluid. The temperature at the interface was set to match the initial temperature of the fluid domain. This boundary condition was used to model the non-isothermal flow of water around the SMP and its effect on the recovery time of the SMP. The lateral walls of these structures were initially set to a reference temperature of 293.15 K. However, due to heat transfer from the surrounding fluid, the temperature of the walls changed over time. The heat transfer process was incorporated into the thermal boundary conditions, resulting in a time-varying temperature profile for the lateral walls. Approach A is similar to approach B, except there is no fluid pressure into microchannels in SMP heat sinks. Therefore, no deformation is expected due to the fluid–structure interaction in approach A. Thus, as Figure 4 exhibits, all the examinations were accomplished with one inlet, and the remaining grooves on the structures’ outer walls were considered outlets. The Reynolds number was 45 at the inlet. Also, it is evident that the mechanical properties of SMPs are temperature-dependent, allowing us to capture material behavior changes as temperature varies.

### 2.4. Thermal Boundary Conditions

Thermal boundary conditions depend on the solving approach. In approach A, convective heat transfer in the structural module is used based on Newton’s law of cooling as follows:

(14) $$q_{0}=h(T_{ext}-T)$$

where h indicates the heat transfer coefficient and is assumed to be 50 $\frac{w}{m^{2}.K}$, T is the temperature of the solid surfaces defined in Figure 5a, and $T_{ext}$ is the reference temperature of 293.15 K. After 80 s, the temperature remains constant until the shape recovery of SMP is entirely performed.

In both approaches, the constant heat flux is prescribed along the top and bottom walls of the structures while the other lateral wall’s initial temperature is set to 293.15 K. Approach B considers the fluid flow in microchannel branches, while the temperature of the moving fluid impacts the structure’s temperature. The heat transfers in structure for approach A’s equation for a solid material with heat flux convection can be represented as follows:

(15) $$\rho C_{p}\frac{\partial T}{\partial t}+\nabla .\left(\lambda \nabla T\right)=Q$$

where Q is the convective heat flux. Also, it is assumed that the radiation of heat transfer is negligible compared to conduction or convection.

## 3. Results and Discussions

**Figure 6:**
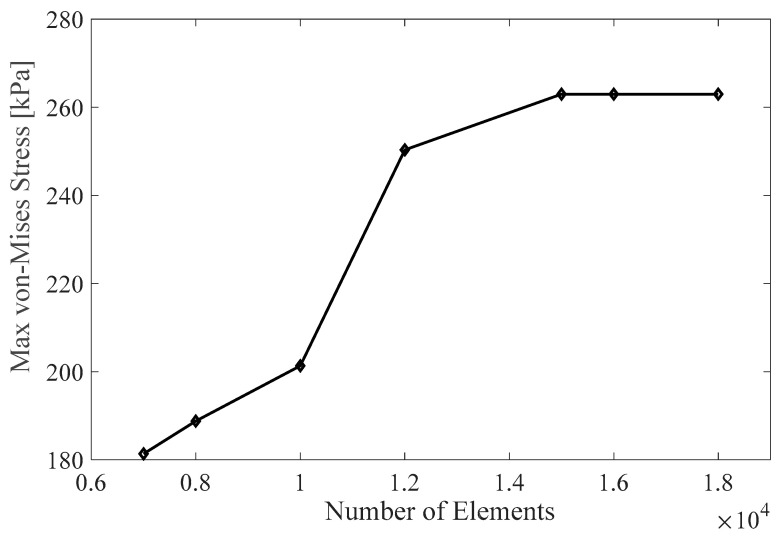
Mesh independence study.

The results are divided into anisotropic and isotropic structures, with six sub-sections examining how the volume fraction, design angle, and usage approaches affect SMP structures’ response. The SMP model used in this study was validated based on the test results in our previous studies [31]. In the following multi-physics simulations, the results were checked independent of the mesh size and time steps. Figure 6 shows the correlation between the mesh size and maximum von Mises stress recovery for an asymmetric anisotropic model. It illustrates the different mesh sizes used, ranging from extra coarse to fine. The maximum element size is 3mm, and the minimum is 0.56 mm, with a growth rate of 1.6. The optimum mesh size for the simulation is approximately 15,000 elements, which was determined through a grid independence study using a tetrahedral mesh.

### 3.1. Anisotropic Structures

The thirteen anisotropic models were designed with various VFs and angles. The wall of the structure that contained the inlet fluid flow was modeled as a fixed boundary condition, and the loading was applied along the structure length (see Figure 4a).

#### 3.1.1. Effect of VF

**Figure 7:**
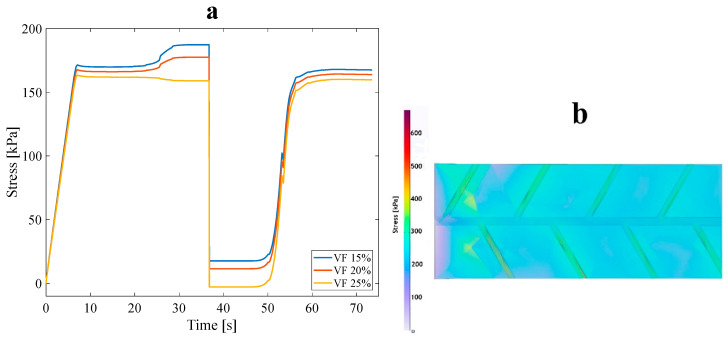
(**a**) Stress recovery cycle for the anisotropic tree-like structure for three VFs: 15%, 20%, and 25% under 3% tensile pre-strain and (**b**) stress distribution for a 15% VF asymmetric structure in the loading state.

**Figure 8:**
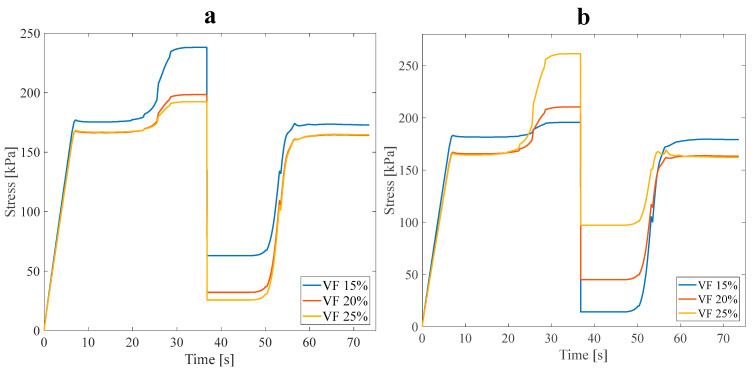
Stress recovery cycle for anisotropic (**a**) asymmetric leaf-like and (**b**) symmetric leaf-like structures for three VFs: 15%,20%, and 25%. Under 3% tensile pre-strain.

Each anisotropic model was numerically designed for three VF values of 15%, 20%, and 25%. All three main anisotropic models were analyzed following approach B’s conditions to investigate the impact of VF on the mechanical and thermophysical properties of the structure. All models were subjected to a 3% tensile pre-strain, and the stress recovery test had already been performed. Figure 7 demonstrates the tree-like structure’s thermomechanical response, where VF is a curve parameter corresponding to approach A (see Figure 2b). Furthermore, Figure 8 illustrates the stress recovery cycle of symmetric and asymmetric structures and their thermomechanical response, loaded with three mentioned VFs under the conditions of approach A.

It is manifestly illustrated in Figure 7 and Figure 8 that at higher values of VF, the necessary stress for applying 3% pre-strain to the structures increases. In particular, increasing the number and volume of sub-branches decreases the stress required to apply a 3% tensile pre-strain in the longitudinal direction of anisotropic structures. This issue is reasonable due to a reduction in the strength of the structures. The α parameter is represented as $\alpha \left(\%\right)=\frac{\sigma^{h}}{\sigma^{l}}\times 100$ where $\sigma^{l}$ is the end of the loading step stress (the required stress for applying the pre-strain) and $\sigma^{h}$ denotes the maximum stress through heating in the stress recovery step [36]. It is expected that, in an ideal SMP, both $\sigma^{h}$ and $\sigma^{l}$ are equal; hence, it is evident that the stress recovery factor should be at least 100% in this case. Based on the classified data in Table 3, as predicted, VF affects the $\alpha \left(\%\right)$. Also, the arrangement and distribution of the sub-branches affect the α and subsequently impact the strength and thermal properties of SMPs. By growing $\alpha \left(\%\right)$, the thermal properties of SMPs are enhanced, and their mechanical properties are reduced. According to the results obtained, different structures have a slight difference between the $\alpha \left(\%\right)$. However, the structures with the highest and lowest α value are selected as the representative structure for approach B. The asymmetric leaf-like structure with 15% VF demonstrated improved mechanical properties; also, all structures with 25% VF had higher thermal properties. Thus, in the following subsection, the four mentioned structures are coupled with fluid within the branches to study the impact of the fluid on stress, shape recovery, and the SMP response time. It is worth mentioning that, in all the models of this section, the geometrical angle between the sub-branches and the main branch was considered at 45°, so the influence of the design angle in this part was ignored.

#### 3.1.2. Effect of Design Angle

**Figure 9:**
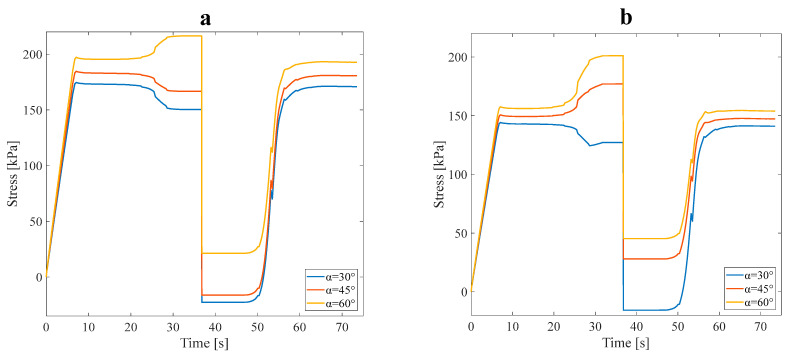
Stress recovery cycle for anisotropic (**a**) symmetric leaf-like and (**b**) asymmetric leaf-like structures for three angles, 30°, 45°, and 60°, under 3% tensile pre-strain.

The following investigates the effect of the angle between the main branch and the sub-branches, which is another design parameter of the structures. The tensile pre-strain is considered at 3%, and this part of the analysis is carried out considering approach A’s conditions. Figure 9 illustrates the stress recovery of SMP in the mentioned conditions for symmetric and asymmetric structures. In this section, two anisotropic asymmetric and symmetric structures were designed with three angles of 30°, 45°, and 60° and subjected to 3% pre-strain tensile loading (see Figure 2a). According to the results of Table 3, the average VF in this subsection is assumed to be 20%.

Investigating the data in Table 4 leads to a fair comparison between the models designed with distinct angles and the α parameter obtained for each of the RVEs separately. Increasing the angle between the main branch and sub-branches decreased the mechanical properties. In approach B, selecting structures with ideal mechanical and thermal properties is required for the numerical analysis. In this regard, structures exhibiting the highest or lowest α value were deemed most suitable due to their superior strength and heat conductivity combination. The asymmetric and symmetric anisotropic structures with a 60° angle were the nearest to the ideal stress recovery (α=100%); after, these structures were employed in the subsequent study. Also, it was anticipated that asymmetric or symmetric anisotropic structures with a 30° angle reveal better thermal properties.

#### 3.1.3. The FSI Simulation and Coupling the Structure with Fluid Flow Effects

**Figure 10:**
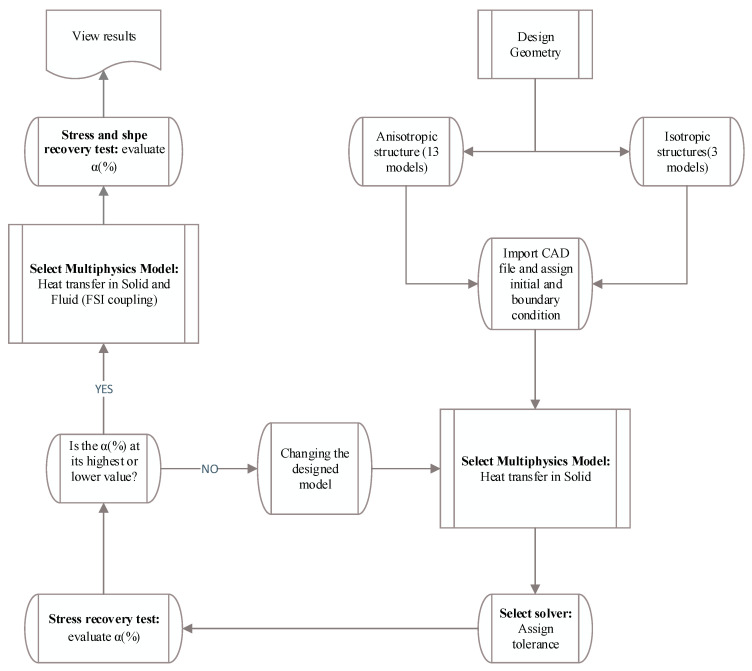
Flowchart of approach B.

In the following, based on the flowchart represented in Figure 10, the selected anisotropic structure corresponding to approach B is discussed. As the flowchart explains, three-dimensional SMP structures were created in SolidWorks for the following two types: anisotropic and isotropic. Anisotropic structures with various VFs and different angles between the main branch and the sub-branches were developed to study the effect of design parameters on SMP’s thermal and mechanical properties. The previously generated computer-aided design (CAD) file was imported into the commercial finite element software, and then material properties and boundary conditions were prescribed. The loading, cooling, fixing, and heating processes were applied to SMPs, and the stress recovery curves were obtained. By calculating the α parameter and choosing its maximum and minimum values, the mathematical optimization process was performed for all 16 structures. The final optimal structure was selected and then numerically investigated regarding approach B. Finally, stress recovery and shape recovery diagrams were plotted by applying appropriate boundary conditions and inlet fluid pressure. Then, the impact of the presence of fluid flow on the heat transfer process in an SMP structure was analyzed.

**Figure 11:**
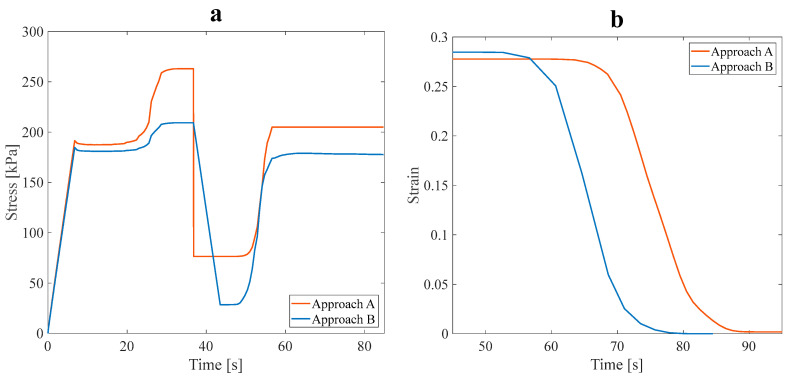
The effect of the fluid in the SME paths of the anisotropic asymmetric leaf-like structure with 15% VF in (**a**) stress recovery and (**b**) shape recovery (during heating step) under 3% tensile pre-strain.

As shown in Figure 11a, the stress recovery test was performed on the optimal models. The tensile pre-strain was assumed to be 3%, and the loading was accomplished in the longitudinal direction. The values of α are evaluated and listed in Table 5.

Based on the values obtained for the α parameter at a 3% pre-strain, it can be inferred that the thermal performance of the SMP structure was enhanced. The impact of the fluid flow on the heat transfer rate in the main branch and sub-branches was analyzed. It was observed that the heat transferred from the fluid to the optimal structure reduced the α value. Fluid flow in the branches of the structure reduced its mechanical strength and properties.

The slight difference of around 5% in α values between the A and B approaches did not significantly affect the mechanical properties. Due to this circumstance, the structure is appropriate for functionalities requiring a faster response time, while mechanical properties are not much reduced. To better distinguish the effect of the fluid on the response time of the optimal SMP structure, the initial conditions were altered to the steps of loading, cooling, unloading, and heating to investigate the shape recovery of the model. To perceive the influence of fluid flow in branches on the thermomechanical response of the model, 3% pre-strain tensile loading was applied.

According to the results shown in Figure 11b, the applied strain, released strain (during the unloading step), and recovered strain can be evaluated. The recovery ratio (at 70 s) in approach B was 79%, and in the A approach, it was 13.04%, occurring at 366.65 K. Therefore, the shape recovery ratio at approach B increased, and the response time reduced. In fact, due to the fluid flow in the branches, the SMP structure recovered its initial shape faster. The fluid flow in the main branch and sub-branches accelerated the shape recovery rate of the SMP structure. Finally, the shape recovery process is performed at a lower temperature (343.5 K). The results present a paramount opportunity and challenge in SMPs. By lowering their work temperature range, SMPs may comply with human body temperature, which could substantially advance their progress and development; this feature makes it an excellent material for numerous applications requiring temperature sensitivity to be considered. Upon completing the preliminary analysis, two anisotropic structures were proposed at an angle of 60° and then analyzed.

**Figure 12:**
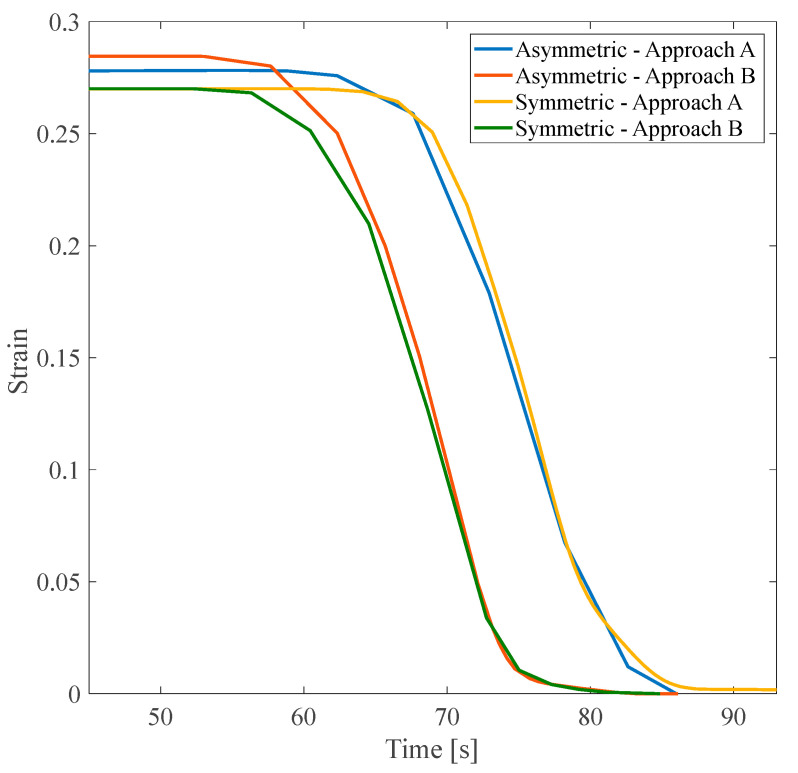
Shape recovery path at the heating step of asymmetric and symmetric structures with the angle of 60° under a 3% tensile pre-strain.

According to Figure 12, it should be noted that although the asymmetric structure had a higher value of the α parameter than that of the symmetric one, the flow and, consequently, heat transfer distribution raised the structure’s temperature faster and accelerated the recovery speed accordingly. In the asymmetric structure, the shape recovery process was performed at a lower temperature, 352.86 K, instead of 367.05 K. Though the symmetric structure has a smaller recovery ratio, it recovered its initial shape faster (i.e., at 353.13 K instead of 357.5 K).

### 3.2. Isotropic Structures

**Figure 13:**
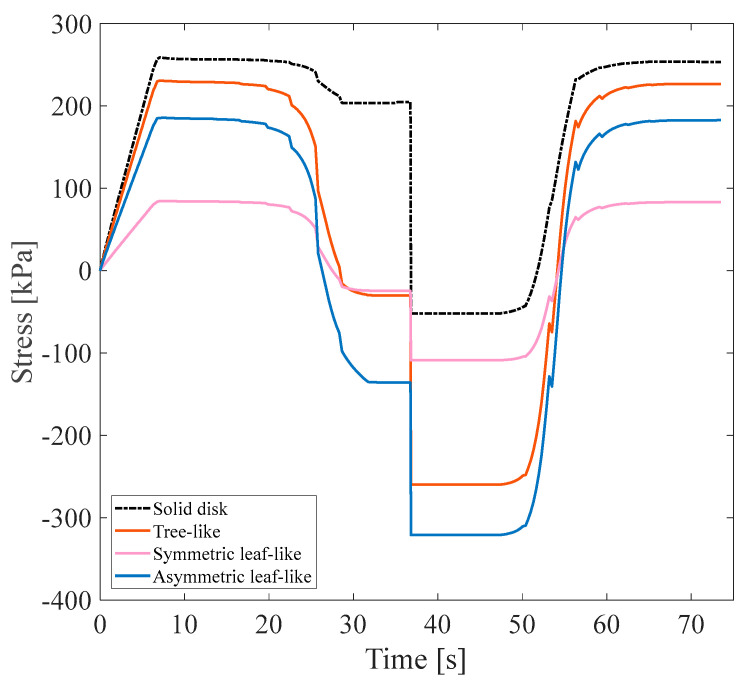
Stress recovery cycle for isotropic structures under 3% tensile pre-strain.

In this subsection, isotropic models were investigated, and the results of stress recovery cycles for the three mentioned isotropic SMPs under 3% pre-strains were demonstrated in Figure 13. 

In this regard, a simple disk without any branch was designed for comparison with other models and subjected to a 3% pre-strain until the presence of branches in the heat transfer rate with approach A was determined. Other designed structures illustrated in Figure 13 included three isotropic models, tree-like, symmetric leaf-like, and asymmetric leaf-like, all with the same VF of 15%. Based on the values obtained for the α parameter according to Table 6, as expected, the simple disk without any branch displayed the highest strength and mechanical properties. It was observed that the highest strength after the solid disk structure was the symmetric leaf-like structure, whose α was 99.40%. Due to the expensive computational cost corresponding to isotropic structures, two structures in terms of mechanical properties and thermal properties were used for the structure–fluid-coupled case.

#### FSI Simulation

**Figure 14:**
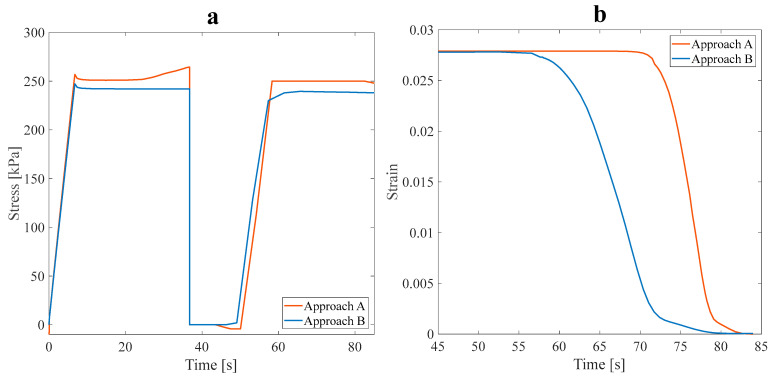
(**a**) Stress recovery cycle, and (**b**) the effect of the fluid in the SME paths for the free-stress-strain recovery of the isotropic symmetric leaf-like structure under a 3% tensile pre-strain.

In this subsection, a symmetric isotropic structure corresponding to the maximum and minimum values of α was determined and numerically studied afterward, considering approach B. The stress recovery cycle in approach B under a 3% tensile pre-strain for the symmetric isotropic model is shown in Figure 14.

Table 7 tabulates the α parameter calculated for both approaches A and B.

Table 7 indicates that the mechanical strength of the optimal SMP with fluid flow within its branches decreased. However, this reduction in mechanical strength is negligible compared to a structure without any fluid. The shape recovery phenomenon was investigated under approach B with 3% pre-strain tensile loading to investigate the effect of the presence of fluid on the thermal properties and response time of the optimal structure. As shown in Figure 14a, the thermomechanical response of structures can be predicted. The motion of fluid in the branch improves stiffness and decreases the amount of the required pre-tensile load to the structure. Therefore, the SMP’s response to temperature variation is further modified.

Regarding mechanical properties, two optimal models regarding anisotropic and isotropic structures can be compared by studying Table 7. The reduction in mechanical properties, such as strength, is evident in the isotropic model; however, this model’s thermal properties improved. For a more thorough investigation of the thermal properties, the shape recovery is shown in Figure 14b. The recovery ratio (at 70s) in approach B for the isotropic symmetric model is 82.38%, and in approach A, it is 25.07%, occurring at 350 K. As for the anisotropic model, the shape recovery ratio rose for approach B, and the response time was reduced. Also, the shape recovery of SMP structures was performed at a lower temperature (344.6 K). Comparing the recovery ratio between two isotropic and anisotropic models demonstrated that the SMP response to temperature variation was considerably faster in the isotropic model than that of the anisotropic one. The application of SMPs in high-frequency actuators and sensors is limited because of their slow response [37]. However, Approach B can overcome this weakness and use it extensively.

**Figure 15:**
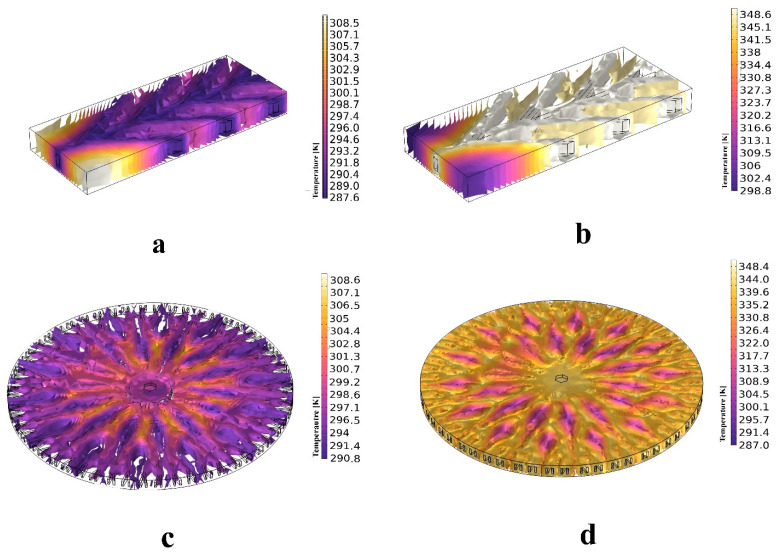
Isothermal contours for the (**a**) anisotropic structure at the cooling step, (**b**) anisotropic structure at the heating step, (**c**) isotropic structure at the cooling step, and (**d**) isotropic structure at the heating step.

Figure 15 presents the 3D isothermal contours of SMP structures at the cooling and subsequent heating stages. The contours highlight temperature distribution during both processes in approach B.

### 3.3. Shape Recovery in Optimal Structures

This subsection focuses on the comprehensive analysis of structures that exhibit the minimum value of parameter α. The significance of this examination lies in the potential to gain an in-depth understanding of the behavior of such structures to optimize them for various applications. This investigation can uncover crucial insights and make informed decisions about research through numerical studies. In this study, only the shape recovery of structures investigates the effect of fluid flow in branches on shape recovery temperature and rate. First, all three anisotropic structures with a 25% VF were studied.

**Figure 16:**
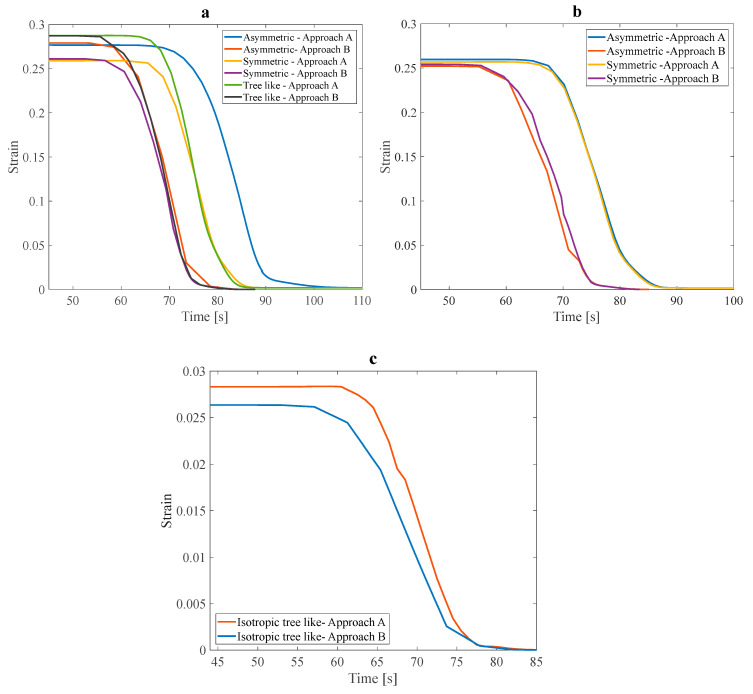
Shape recovery path of (**a**) three anisotropic structures with 25% VF, (**b**) two anisotropic structures with a design angle of 30°, and (**c**) a tree-like isotropic structure under a 3% pre-strain.

The inlet water pressure was 5 Pa, and the tensile pre-strain was 3%. The estimated heating time was 40 s. As shown in Figure 16a, in all three mentioned structures, with a linear increase in temperature from 40 s to about 100 s, recovery was faster in the structure that contained fluid flow. Through this research, it was discovered that elevating the slope of the recovery diagram in fluid-containing structures leads to more rapid and complete shape recovery. Furthermore, this alteration also impacts the recovery temperature, which is essential in optimizing the shape recovery process for various applications. Reducing the working temperature range of SMPs is a great way to improve their suitability for various applications.

The structures shown in Figure 16b have the same design angle, α parameter, and VF. The only difference between these two structures is how the fluid is distributed in the structure. Slight variations in fluid distribution within a structure can significantly impact the overall recovery behavior and performance of SMPs. Additionally, it was observed in the asymmetric structure that the temperature range required for the shape recovery process to occur was significantly reduced. The following structure to be studied in this section is the isotropic tree-like structure. Figure 16c shows a noticeable difference in the recovery slope and total shape recovery duration depending on whether the fluid is present. This observation underscores the significant impact that fluid can have on the recovery process. To more accurately investigate the effect of fluid on the recovery temperature, it is necessary to compare the initial recovery temperature and the recovery temperature in the presence of fluid in the above structures. As shown in Table 8, in general, with fluid flow in the branches embedded in the structures and heat transfer through these networks, the recovery temperature can be improved, and the working range of the temperature of the SMPs can be reduced.

Based on the data presented in Table 8, structures that have fluid flow in their embedded branches recover faster. Additionally, complete recovery occurred at a lower temperature for all the studied SMP structures. Among the anisotropic structures, those with an angle design of 30° with a lower α parameter in approach A showed the best reduction in recovery temperature in approach B. These results show that, in general, the design parameters of the structure, such as the angle between the main branch and the sub-branches, can improve convection heat transfer in the structures, make this structure more sensitive to temperature changes, and have a faster recovery. It is essential to remember that when assessing a structure, it is not only enough to consider the final recovery temperature, but the recovery ratio should also be involved in this comparison for a more accurate assessment. The recovery ratio varies significantly between 10% and 60%, depending on the type of structure. The most significant difference in the recovery ratio, representing the recovery speed, was in the isotropic symmetric leaf-like and anisotropic 60° asymmetric leaf-like structures. Isotropic structures are a better option for high-temperature sensitivity and faster recovery due to their uniform heat and fluid flow distribution.

## 4. Summary and Conclusions

This research proposes a new process to facilitate the development of SMP’s thermal performance and the simultaneous retention of their mechanical properties. Sixteen three-dimensional structures with various topologies were generated and exploited to investigate the heat transfer effect via fluid flow in microchannel networks (branches) and its impact on the thermal response and shape recovery of SMPs. Since temperature-sensitive SMPs have attracted more researchers due to their vast applications, a faster shape recovery has become significant, especially in biomechanics and operating them as sensors and actuators. The designed structures were analyzed to improve the shape’s recovery speed. The recovery ratio and recovery parameter (α) were computed for all models. Based on the results between the two classes of isotropic and anisotropic models, isotropic models had a higher recovery speed and, under coupling with the fluid, performed better for the heat transfer and thermal response.

On the other hand, the effect of design parameters on anisotropic structures provided exciting results. Following the results, the effect of the VF of networks, which is calculated by the number of main branches and sub-branches, on the strength and mechanical properties of the structures was investigated, and structures with a lower VF had more satisfactory thermal properties. The results show that increasing the angle between the main branch and sub-branches can enhance the thermal properties of a structure. This modification can result in accelerated shape recovery. In short, the following results were obtained:

- The recovery ratio and parameter (α) were computed for all isotropic and anisotropic structures.
- Anisotropic structures with a 25% VF demonstrated the highest recovery ratio when coupled with water.
- Anisotropic structures with a 30° angle between the main branch and sub-branches exhibited a much lower temperature for shape recovery in the coupled state with the fluid.
- Isotropic structures have a higher recovery speed and exhibit better heat transfer and thermal response when coupled with fluid.
- The effect of the VF of networks on the strength and mechanical properties of the structures was investigated, and structures with a lower VF demonstrated more satisfactory thermal properties.
- Decreasing the angle between the main branch and sub-branches can enhance the thermal properties of a structure.
- The implemented methods in this study did not cause a significant decrease in the mechanical properties of the structures.

Investigating fluid flow in microchannel networks highlights the importance of considering heat transfer impacts when designing and optimizing SMP structures. By comprehending how fluid flow influences the thermal response of SMPs, researchers can develop more efficient and effective systems for applications that require rapid shape recovery. This study demonstrates that the proposed process does not significantly compromise the mechanical properties of SMP structures. This is crucial as maintaining mechanical integrity is essential for applying SMPs, especially in load-bearing scenarios.

## Figures and Tables

**Table 1:** Material parameters calibrated for experiments [35].

**Viscoelastic Properties**						
Energy factor ($b_{z}$)	5.862759 522	101.590 4875	1.379170 492	0.095327 903	0.004518 184	0.010219 807
Relaxation time ($t_{z}$)	0.00141	0.0000428	0.0208	0.74	46	100,000
**Hyperelastic Properties**						
Strain energy potential			Neo-Hookean			
Incompressible		µ (Pa) 1,919,999.996 4				
			WLF			
	$T_{ref}({}^{\circ})$		$C_{1}$		$C_{2}$	
	80		6.9		87.9	
			Silicone			
Strain energy potential		Neo-Hookean				
Incompressible		µ (Pa) 400,000				

**Table 2:** Material properties of the fluid (water).

Material Properties	Water (273.15K≤T≤373.15)
Density (ρ) $\left[\frac{\mathrm{kg}}{m^{3}}\right]$	$432.25+4.96T-0.01T^{2}+1.03\times 10^{-5}T^{3}$
Thermal conductivity (λ) $\left[\frac{W}{mK}\right]$	$-0.86+8.94\times 10^{-3}T-1.58\times 10^{-5}T^{2}+7.97\times 10^{-9}T^{3}$
Specific heat capacity (C_p_) $\left[\frac{J}{Kg.K}\right]$	$12010.14-80.40T+0.30T^{2}-5.38\times 10^{-4}T^{3}+3.62\times 10^{-7}T^{4}$
Dynamic viscosity $\left(\mu \right)\;[Pa.s]$	$1.37-0.02T+1.36\times 10^{-4}T^{2}-4.64\times 10^{-7}T^{3}+8.90\times 10^{-10}T^{4}-9.07\times 10^{-13}T^{5}+3.84\times 10^{-16}T^{6}$

**Table 3:** Values of $\alpha \left(\%\right)$ for anisotropic structures under 3% tensile pre-strain.

Anisotropic Structures	VF of Branches	$\sigma^{l}$ (kPa)	$\sigma^{h}$ (kPa)	α (%)
	15%	169.952	168.0	98.85
Tree-like	20%	166.271	164.290	98.80
	25%	162.105	160.126	98.77
	15%	182.0	180.0	98.90
Symmetric leaf-like	20%	166.0	164.0	98.79
	25%	164.485	162.291	98.66
	15%	175.411	173.610	98.97
Asymmetric leaf-like	20%	166.970	165.0	98.82
	25%	166.490	164.607	98.86

**Table 4:** Values of $\alpha \left(\%\right)$ for various structures under 3% tensile pre-strain

Anisotropic Structures	Angle of Branches	$\sigma^{l}$ (kPa)	$\sigma^{h}$ (kPa)	α (%)
	30°	173.328	171.151	98.74
Symmetric leaf-like	45°	183.203	180.922	98.75
	60°	195.546	193.280	98.84
	30°	143.124	141.330	98.74
Asymmetric leaf-like	45°	149.434	147.769	98.88
	60°	175.411	173.610	98.94

**Table 5:** Values of $\alpha \left(\%\right)$ for the optimal anisotropic structure under 3% tensile pre-strain.

Anisotropic Structure	Approaches	$\sigma^{l}$ (kPa)	$\sigma^{h}$ (kPa)	α (%)
Asymmetric leaf-like (VF15% and angle 45°)	B	184.736	170.658	93.37
	A	175.411	173.610	98.97

**Table 6:** Values of $\alpha \left(\%\right)$ for various isotropic structures under 3% tensile pre-strain.

Isotropic Structures	$\sigma^{l}$ (Pa)	$\sigma^{h}$ (Pa)	α (%)
Symmetric leaf-like	83.7	83.2	99.40
Asymmetric leaf-like	183	185	98.91
Tree-like	223	227	98.69
Solid disk	254	253.6	99.84

**Table 7:** Values of $\alpha \left(\%\right)$ for the optimal isotropic structure under 3% tensile pre-strain.

Isotropic Structure	Approaches	$\sigma^{l}$ (kPa)	$\sigma^{h}$ (kPa)	α (%)
	B	247.229	239.492	96.87
Symmetric leaf-like				
	A	83.7	83.2	99.40

**Table 8:** Comparison of initial recovery temperature and recovery temperature with fluid flow. The recovery ratio was evaluated at the same time for approaches A and B.

Geometric Design	Structure Name	Shape Recovery Temperature (Approach B) [K]	Initial Shape Recovery Temperature [K]	Recovery Ratio (Approach B) %	Recovery Ratio (Approach A) %
	Asymmetric leaf-like/VF25%	351.32	358.44	79.29	9.74
	Symmetric leaf-like/VF25%	351.81	367.31	73.82	20.01
	Tree-like/VF25%	357.05	367.80	73.81	5.78
**Anisotropic**	Asymmetric leaf-like/VF15%	343.5	366.65	79.06	13.04
	Symmetric leaf-like/angle 30°	351.36	368.23	66.69	11.65
	Symmetric leaf-like/angle 60°	353.13	367.05	87.45	19.23
	Asymmetric leaf-like/angle 60°	352.86	355.59	65.70	35.63
	Asymmetric leaf-like/angle 30°	353.42	370.53	64.61	10.87
**Isotropic**	Symmetric leaf-like	344.6	350	82.38	25.08
	Tree-like	347.32	352.65	62.65	43.89

## Data Availability

Data is contained within the article.
